# Supplementary material for: Reproductive Pattern of Parous Women and the Risk of Cancer in Later Life
Source: Cancers (Basel). 2021 Jul 24;13(15):3731. doi: 10.3390/cancers13153731 (PMC8345127; doi:10.3390/cancers13153731)
Supplement: Supplementary file 1 [file cancers-13-03731-s001.zip › cancers-1283081-supplementary.pdf]

Reproductive Pattern of Parous Women and The Risk of Cancer in Later Life

Zahra Pasdar, Neil W Scott, Lisa Iversen, Philip C Hannaford, Phyo K Myint and Sohinee Bhattacharya

Table S1. ICD–9 and 10 codes used to identify and categorise cancer sites.

Cancer Site	ICD-9 Codes	ICD–10 Codes
Breast	174	C50
Uterus (body)	182	C54
Uterus (cervix)	180	C53
Carcinoma in Situ	233.1	D06
Ovary	183	C56
Skin	172, 173	C43 to C44
Respiratory	160–165	C30 to C39
Gastrointestinal	150–159	C15 to C26
Other cancers (includes any other malignant neoplasm not classified in any of the above)	140–209 excluding the codes already specified above	Any other C or D code not classified above

Table S2. Descriptive statistics for subgroup analyses by cancer site (breast/gynaecological cancers): reproductive variables by case-control status.

Reproductive pattern	Breast		Uterus		Uterine Cervix		Ovary		Carcinoma in Situ	
	N(Ca)	N(Co)	N(Ca)	N(Co)	N(Ca)	N(Co)	N(Ca)	N(Co)	N(Ca)	N(Co)
Total number of pregnancies										
1 (reference)	305	278	29	33	38	32	32	28	302	248
2	457	490	45	43	57	54	28	45	697	611
3+	449	443	42	40	57	66	44	31	579	719
Total	1211	1211	116	116	152	152	104	104	1578	1578
No. of pregnancies of normal duration										
1 (reference)	337	310	29	34	45	38	34	30	353	340
2	531	537	56	53	69	57	36	50	814	731
3+	343	364	31	29	38	57	34	24	411	507
Total	1211	1211	116	116	152	152	104	104	1578	1578
Cumulative time pregnant (weeks)										
≤50	312	286	29	33	39	32	32	38	320	276
51–100	485	505	52	47	67	52	33	46	779	689
101+	270	299	23	25	31	42	26	18	361	428
Total	1067	1090	104	105	137	126	91	92	1460	1393
Age at first delivery										

≤20	280	231	30	24	28	67	17	15	435	685
21-25	526	529	62	60	80	50	40	46	575	590
26-30	266	310	14	26	25	28	30	32	382	232
31+	136	139	10	5	18	7	17	10	185	70
Total	1208	1209	116	115	151	152	104	103	1577	1577
Interpregnancy interval										
No subsequent pregnancy	350	310	34	37	50	39	37	31	361	317
<3 years	486	535	50	44	55	58	37	42	664	630
>3 years	278	268	24	29	32	41	21	23	385	419
Total	1114	1113	108	110	137	138	95	96	1410	1366

Table S3: Descriptive statistics for subgroup analyses by cancer site (non-gynaecological cancers): reproductive variables by case-control status.

Reproductive pattern	Skin		Respiratory		Gastrointestinal		Other Cancers	
	N(Ca)	N(Co)	N(Ca)	N(Co)	N(Ca)	N(Co)	N(Ca)	N(Co)
No of pregnancies of normal duration								
1 (reference)	91	88	133	103	196	176	418	386
2	126	127	151	164	218	248	544	608
3+	127	129	192	209	251	241	634	602
Total	344	344	476	476	665	665	1596	1596
Total no. of pregnancies								
1 (reference)	104	94	145	114	212	186	457	424
2	137	143	173	181	241	269	637	675
3+	103	107	158	181	212	210	502	497
Total	344	344	476	476	665	665	1596	1596
Cumulative time pregnant (weeks)								
≤50	97	90	137	105	203	179	423	395
51–100	133	135	162	158	222	260	582	623
101+	87	89	116	137	168	172	391	418
Total	317	314	415	400	593	611	1396	1436
Age at first delivery								
≤20	66	70	91	121	118	98	332	355
21–25	124	136	218	211	287	276	738	674
26–30	96	90	112	100	167	181	346	377
31+	58	48	55	44	93	109	177	187
Total	344	344	476	476	665	664	1593	1593
Interpregnancy interval								
No subsequent pregnancy	101	105	143	119	226	203	474	452
<3 years	140	140	185	185	255	266	652	627
>3 years	77	75	110	141	150	165	347	423
Total	318	320	438	445	631	634	1473	1502
